# Autoantibodies neutralizing type 1 interferons in two cohorts of people with HIV

**DOI:** 10.70962/jhi.20250179

**Published:** 2026-01-30

**Authors:** Julie A. Jensen, Nanna Mørk, Martin Tolstrup, Ole Søgaard, Thomas Dalhuisen, Antonio Rodriguez, Rebecca Hoh, Michael J. Peluso, Steven G. Deeks, Jean-Laurent Casanova, Trine H. Mogensen

**Affiliations:** 1 Department of Biomedicine, Aarhus University, Aarhus, Denmark; 2 Department of Infectious Diseases, Aarhus University Hospital, Aarhus, Denmark; 3 Center for Immunology of Viral Infections, Aarhus University, Aarhus, Denmark; 4 Department of Clinical Medicine, Aarhus University, Aarhus, Denmark; 5 Division of HIV, Infectious Diseases, and Global Medicine, University of California, San Francisco, CA, USA; 6 Laboratory of Human Genetics of Infectious Diseases, Institut National de la Santé et de la Recherche Médicale, Necker Hospital for Sick Children, Paris, France; 7 St. Giles Laboratory of Human Genetics of Infectious Diseases, The Rockefeller University, New York, NY, USA; 8 Howard Hughes Medical Institute, New York, NY, USA; 9 Department of Pediatrics, Necker Hospital for Sick Children, Paris, France; 10 Imagine Institute, Paris Cité University, Paris, France

## Abstract

Autoantibodies (autoAbs) neutralizing type I interferons (IFN-I) markedly increase the risk of severe manifestations of some viral diseases. The prevalence of autoAbs neutralizing at least one IFN-I, specifically IFN-α2, IFN-β, and IFN-ω, in individuals from the Swiss human immunodeficiency virus 1 (HIV-1) cohort is largely similar to French age-matched healthy controls, with around 1% in adults by age 65 having neutralizing IFN-I autoAbs and rising thereafter. We analyzed samples from Danish individuals with HIV, including antiretroviral therapy (ART) treated (*n* = 260) and ART-naive (*n* = 58) individuals, aged 18–79 (mean age 46.6 years, SD ± 11.1 years), and American individuals with HIV, all of whom were ART-naive (*n* = 292) aged 20–76 (mean age 41.7 years, SD ± 10.1 years). In the Danish cohort, a total of 2/318 (0.63%) individuals had autoAbs neutralizing 1 ng/ml IFN-ω, whereas 1/318 (0.31%) had autoAbs neutralizing 200 pg/ml IFN-ω. In the American cohort, 2/292 (0.68%) individuals had autoAbs neutralizing IFN-ω at 1 ng/ml. Combining both cohorts, the overall prevalence of autoAbs neutralizing IFN-ω was 5/610 (0.82%). Longitudinal samples were available for two individuals with IFN-ω autoAbs: One displayed persistently detectable autoAbs over time, whereas the other showed a decline in neutralizing activity after 2 years. Two out of five individuals with IFN-I neutralizing autoAbs had a history of IFN-α treatment for chronic hepatitis C virus (HCV) infection. Individuals with IFN-I neutralizing autoAbs shared a history of viral infections, including mucocutaneous herpesvirus infections, zoster, and human papillomavirus disease. Collectively, the prevalence of IFN-I neutralizing autoAbs in people with HIV, whether ART-treated or ART-naive, did not significantly differ from that in the general population. Whereas hepatitis C and IFN therapy may contribute to the development of autoAbs to IFN-I, these autoAbs in turn seem to predispose to mucocutaneous herpesvirus infections and HPV-related neoplasia.

## Teaser

Here, Jensen and colleagues studied type I interferon (IFN-I) neutralizing autoAbs in people with HIV, and found that the prevalence does not significantly differ from that in the general population. While hepatitic C and IFN therapy likely contribute to the development of IFN-I autoAbs, these may predispose to mucocutaneous herpesvirus infections and HPV-related neoplasia.

## Introduction

Viral infections are associated with vast interindividual clinical variability, as manifestations of the same virus can range from asymptomatic infection to lethal disease. While rare inborn errors of type I interferon (IFN-I) immunity have been implicated from 2003 onward, the more common autoantibodies (autoAbs) neutralizing IFN-I have been studied from 2020 onward (1). Besides a case of disseminated zoster reported between 1981 and 1984 (2, 3), IFN-I autoAbs were considered to be clinically silent until the COVID-19 pandemic, during which they were shown to underlie 15% of hypoxemic COVID-19 pneumonia cases and 20% of fatal COVID-19 cases (4, 5). Since these discoveries, IFN-I neutralizing autoAbs have been shown to underlie several other severe viral diseases, including: ∼5% of severe influenza pneumonia (6), ∼20% of hypoxemic Middle East respiratory syndrome pneumonia (7), one-third of adverse reactions to live-attenuated yellow fever vaccine (8), ∼40% of West Nile virus encephalitis (9), ∼10% of tick-borne encephalitis (10), and most cases of rare severe arboviral diseases (11). In a large cohort of ∼35,000 healthy individuals, IFN-I neutralizing autoAbs were detected in 0.3–1% of adults under the age of 65 years and in 4–7% of adults over 65 years (5, 11). IFN-I neutralizing autoAbs can be seen as “autoimmune phenocopies” of inborn errors of IFN-I immunity, thereby predisposing individuals to severe viral infections (1, 12, 13). The full spectrum of viral infections driven by IFN-I neutralizing autoAbs may be broader than currently recognized. While these autoAbs are found in a growing range of inborn errors of thymic tolerance to self, including the prototypical autoimmune polyglandular syndrome type 1 (APS-1) (14) and related conditions (15), they are also more common in patients with various autoimmune conditions of elusive or incomplete etiology (13, 16).

Recently, there has been growing interest in examining the prevalence of IFN-I neutralizing autoAbs in individuals with human immunodeficiency virus 1 (HIV-1). A recent study by Hale and associates leveraged a large number of consecutive samples from the Swiss HIV Cohort, consisting of virally suppressed antiretroviral therapy (ART)–treated individuals living with HIV. Participants were screened for the presence, persistence, and development of IFN-I neutralizing autoAbs. Among nearly 2,000 people with HIV, aged 65 or above (age distribution: 65–69 years [*n* = 857], 70–79 years [*n* = 831], 80–89 years [*n* = 183], and 90–94 years [*n* = 5]) at time of sampling, 1.17% developed IFN-I neutralizing autoAbs against at least one IFN-I, based on concentrations of 10, 1, or 0.2 ng/ml for IFN-α2 and IFN-ω, and 1, 0.2, or 0.04 ng/ml for IFN-β. This prevalence is comparable to that reported in a large study on the general population, where IFN-I autoAbs were detected in ∼1% under 70 years, 2.3% between 70 and 80, and 6.3% above 80 years (5). Longitudinal testing of autoAbs-positive individuals in the Swiss HIV Cohort study over an average of 20.2 years prior to the initial sample revealed a median onset of autoAbs development of 63 years, with age ranges spanning from 45 to 80 years (17). The major findings of the study of the Swiss HIV Cohort were that neutralizing IFN autoAbs arise and do not disappear over time. They also diversify, the neutralization of an IFN preceding that of another. Moreover, the authors suggested that prior viral infection, including cytomegalovirus (CMV) status, IFN treatment for viral hepatitis B or C, or autoimmune diseases might predispose to the development of IFN autoAbs (13, 17).

Overall, the mechanisms and causes underlying the development of IFN-I neutralizing autoAbs remain unresolved in most individuals, especially in the elderly. To better characterize the natural history of these autoAbs, we tested two cohorts of individuals with HIV-1 infection. There are several key questions regarding the impact and development of IFN-I neutralizing autoAbs in the context of HIV-1 infection. One is whether chronic HIV-1 infection, with or without viremia, predisposes or induces IFN-I neutralizing autoAbs. A second is whether IFN-I neutralizing autoAbs may contribute to immune exhaustion and opportunistic infections in this immune-dysregulated pathological context. In the present study, we sought to answer these questions by investigating different cohorts of viremic ART-naive and virally suppressed ART-treated people with HIV, examining their medical history of opportunistic viral infections and virus-related cancer, and correlating this to circulating IFN-I neutralizing autoAbs over time.

## Results

### Demographics of two adult cohorts with HIV-1-infection

**Figure 1.**
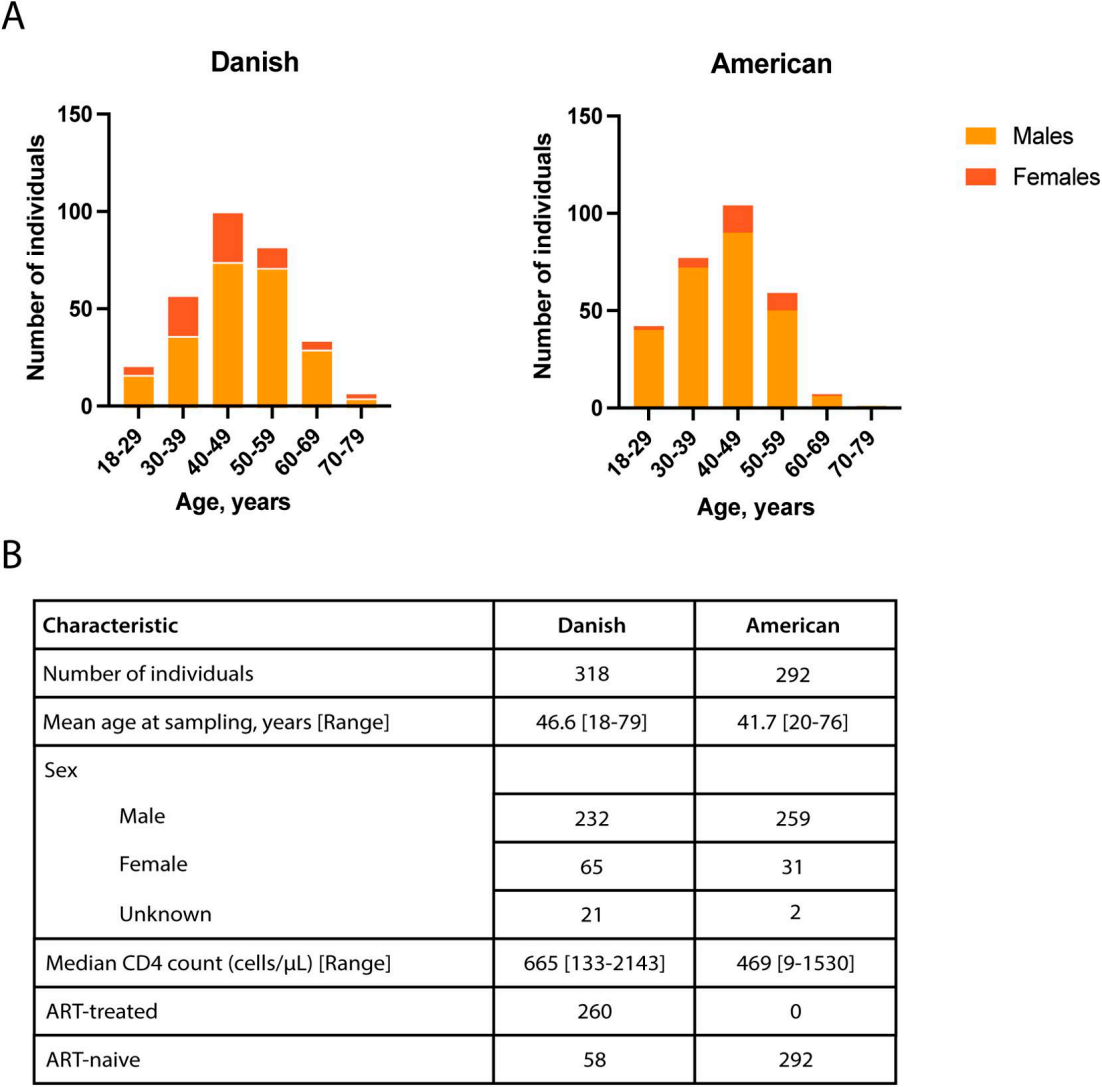
**Baseline characteristics of the study population.** The cohorts consisting of individuals from a Danish cohort (*n* = 318) and an American cohort (*n* = 292) diagnosed with HIV. Individual data at the time of inclusion for blood samples are shown. **(A)** Stacked bar charts showing the age distribution of individuals in the Danish and American cohorts, stratified by sex. Age was available for 296/318 (93%) individuals in the Danish cohort and 292/292 (100%) individuals in the American cohort. **(B)** Summary table detailing characteristics from each cohort, including total number of individuals, mean age, sex distribution, CD4^+^ T cell counts, and ART-treatment status. The Danish cohort included both ART-treated and ART-naive individuals, whereas all individuals in the American cohort were ART-naive.

Adults diagnosed with HIV-1 from two independent cohorts were included: a Danish cohort from Aarhus University Hospital (AUH), Denmark, and an American cohort from the University of California, San Francisco (UCSF), San Francisco, CA, USA. In total, 657 plasma samples were obtained, of which 365 plasma samples were from 318 Danish individuals (including 47 longitudinal samples), and 292 plasma samples were from 292 American individuals. The exact age at plasma collection was available for 296/318 (93.1%) individuals in the Danish cohort and for all individuals in the American cohort (Fig. 1 A). The age of the individuals ranges from 18 to 79 years in the Danish cohort, with a mean (±SD) age at plasma sampling of 47.3 (±11.2) years for men and 44.0 (±10.5) years for women, resulting in an overall mean age of 46.6 (±11.1) years. In the American cohort, the age ranged from 20 to 76 years at plasma sampling, and the mean age was 41.2 (±10.2) years for men and 45.0 (±8.9) for women, with an overall mean age of 41.7 (±10.1) years. Most individuals, 282/296 (95.3%) in the Danish cohort and 288/292 (98.6%) in the American cohort were below the age of 65—an age group associated with a relatively low prevalence of IFN-I neutralizing autoAbs in the general population (5). Sex was known for 297/318 (93.4%) individuals in the Danish cohort, of which 232/297 (78.1%) were male and 65/297 (21.9%) were female. In the American cohort, sex was known for 290/292 (99.3%) individuals, of which 259/290 (89.3%) were male and 31/290 (10.7%) were female. CD4^+^ T cell counts ranged from 133 to 2,143 cells/μl in the Danish cohort, with a median count of 665 cells/μl based on data from 208/318 (65.4%) individuals. In the American cohort, CD4^+^ T cell counts ranged from 9 to 1,530 cells/μl, with a median of 469 cells/μl (data available for all 292 individuals). Regarding treatment status, 260/318 (81.8%) of the Danish individuals were on ART, while 58/318 (18.2%) were ART-naive. In contrast, all 292/292 (100%) American individuals were ART-naive (Fig. 1 B).

### Presence of IFN-I neutralizing autoAbs in adults with chronic HIV-1 infection

**Figure S1.**
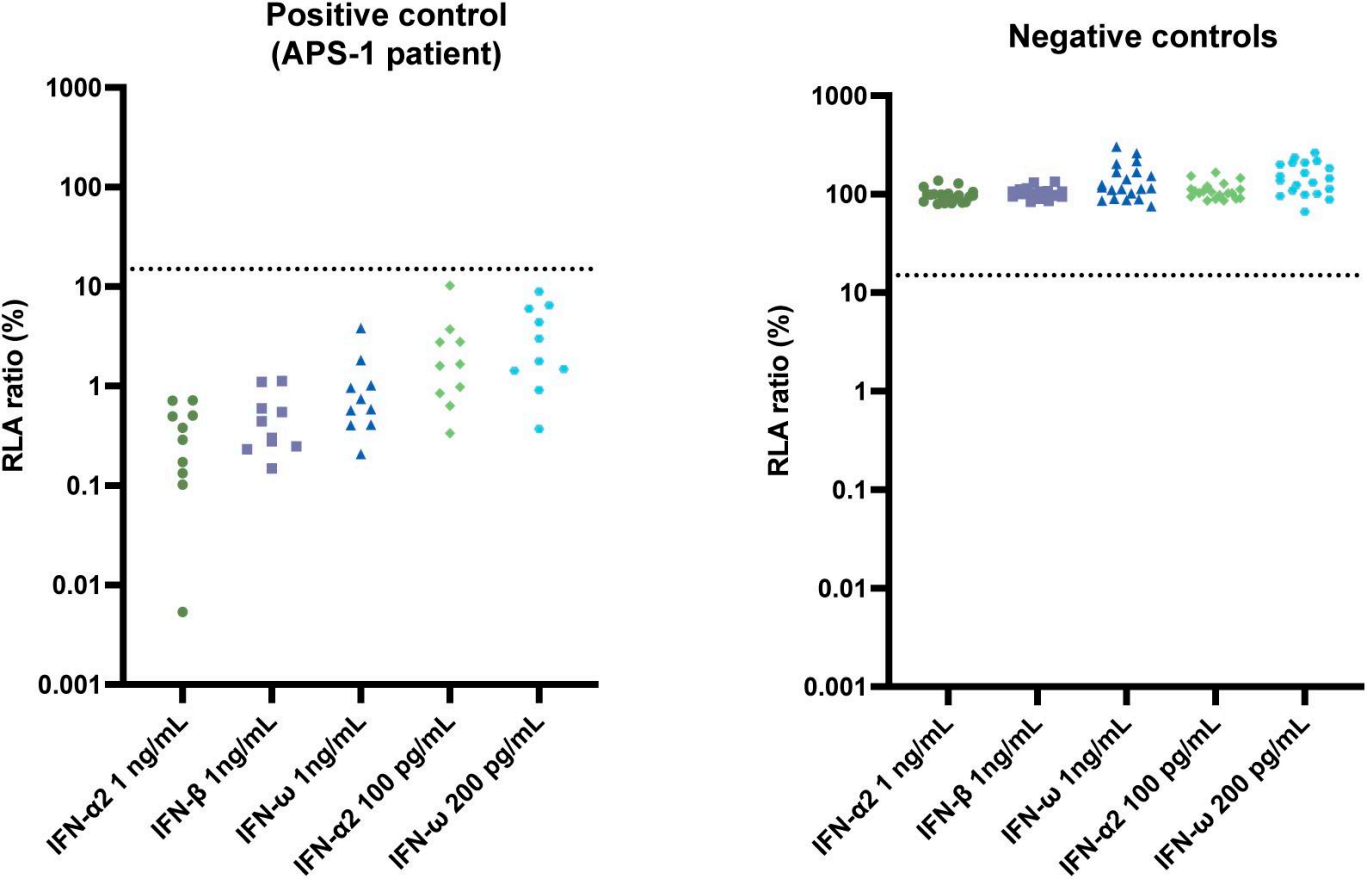
**Longitudinal measurements of IFN-I neutralizing autoantibodies in participants P4 and P5.** Measurement of IFN-I neutralizing autoAbs plotted as normalized relative luciferase activity after baseline correction (RLA ratio) for an IFN-I autoAbs-positive APS-1 patient, demonstrating neutralization against IFN-α, -β, and -ω at all concentrations (left) and for two IFN-I neutralizing autoantibody (autoAbs) negative controls run on each plate (right).

**Figure 2.**
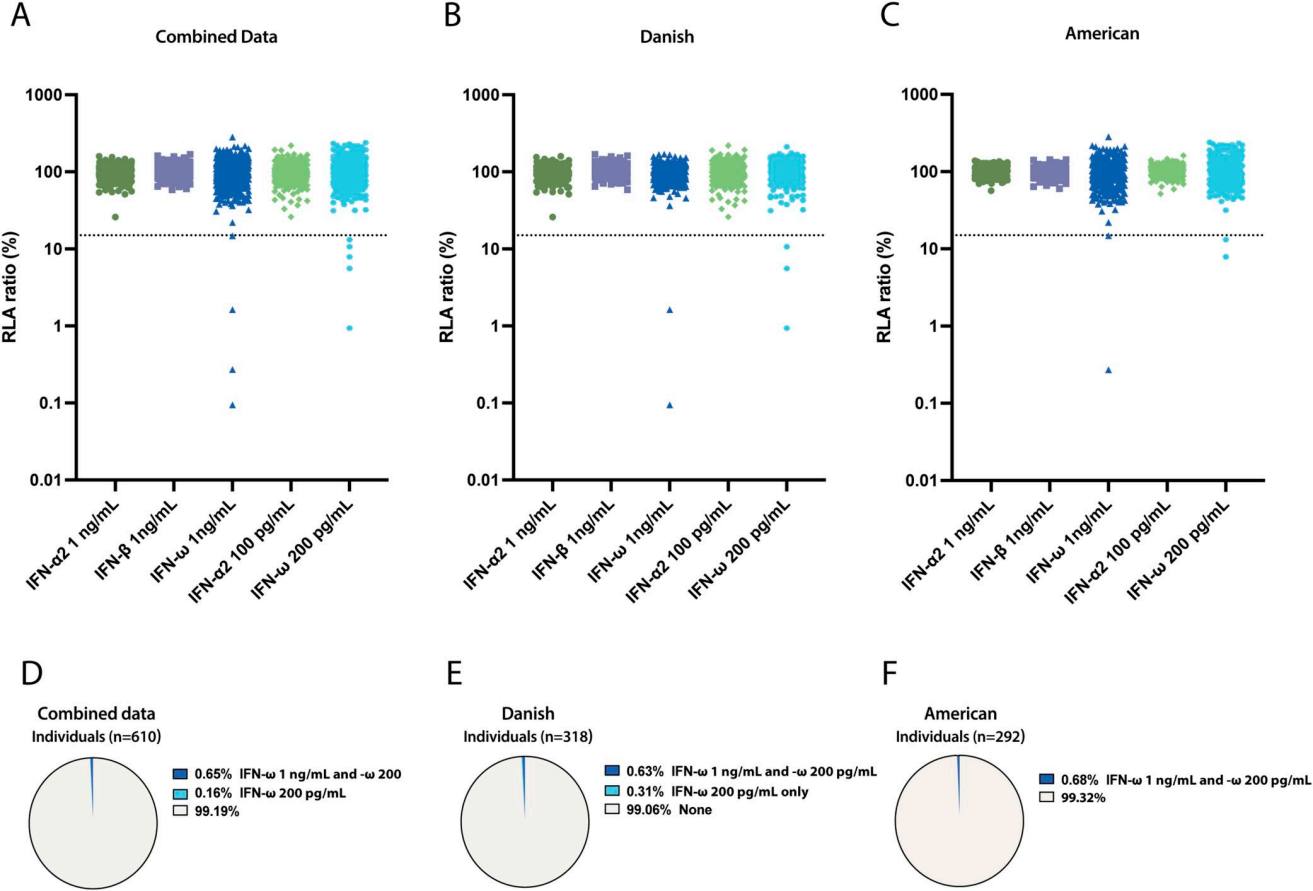
**Capacity of plasma samples from individuals with HIV to neutralize** **IFN-I** **.** Plasma samples from individuals with HIV-1 infection tested for their ability to neutralize five different conditions of IFN-I by applying plasma to HEK293T cells for 16 h. Neutralization was defined as a response below 15% of normalized relative luciferase activity (RLA ratio), represented as a dotted line in each panel. All samples were tested once in a dual luciferase assay. **(A–C)** RLA ratio (%) plotted, with values below 15% indicating neutralization. Each panel represents samples tested at the specific IFN-I condition as specified. **(D–F)** Pie charts summarizing the percentage and combination of IFN-I conditions neutralized per individual plasma sample.

IFN-I neutralization was assessed using a luciferase-based human embryonic kidney (HEK)-293T cell assay (5, 9). Unglycosylated IFN-α2 was tested at concentrations of 1 ng/ml and 100 pg/ml, glycosylated IFN-β at 1 ng/ml, and unglycosylated IFN-ω at 1 ng/ml and 200 pg/ml. As a positive control, we used plasma from a patient diagnosed with APS-1 known to have autoAbs against all tested IFN-I conditions, which was confirmed in our assay. Plasma samples from two healthy donors lacking IFN-I neutralizing autoAbs were used as negative controls (Fig. S1). Across both cohorts (*n* = 610), IFN-I neutralizing autoAbs against the five different IFN-I subtypes and/or concentrations were measured. We only detected neutralization against IFN-ω, where four individuals exhibited neutralization against both IFN-ω at 1 ng/ml and 200 pg/ml and one individual only against IFN-ω at 200 pg/ml (Table 1 and Fig. 2, A–C). This resulted in the presence of IFN-I neutralizing autoAbs in at least one tested condition in 5/610 (0.82%) individuals. In the Danish cohort, 2/318 (0.63%) individuals had neutralizing autoAbs against both IFN-ω 1 ng/ml and 200 pg/ml, while 1/318 (0.31%) had neutralizing autoAbs only against IFN-ω at 200 pg/ml. In the American cohort, 2/292 (0.68%) individuals had IFN-I neutralizing autoAbs against both IFN-ω 1 ng/ml and 200 pg/ml (Fig. 2, D–F). We did not detect IFN-I neutralizing autoAbs against either IFN-α2 or IFN-β in any of the individuals tested.

**Table 1.** Summary of neutralizing samples per IFN-I condition tested

Sample category	IFN-α2 1 ng/ml	IFN-β 1 ng/ml	IFN-ω 1 ng/ml	IFN-α2 100 pg/ml	IFN-ω 200 pg/ml	Individuals with neutralizing autoAbs
Danish cohort, *N* (%)	0/318 (0.00)	0/318 (0.00)	2/318 (0.63)	0/318 (0.00)	3/313^a^ (0.96)	3/318 (0.94)
American cohort, *N* (%)	0/292 (0.00)	0/292 (0.00)	2/292 (0.68)	0/292 (0.00)	2/292 (0.68)	2/292 (0.68)
Combined cohorts, *N* (%)	0/610 (0.00)	0/610 (0.00)	4/610 (0.65)	0/610 (0.00)	5/605 (0.83)	5/610 (0.82)

a Five samples excluded based on reduced transfection efficiency.

### Comparison of demographics and laboratory data between IFN-I autoAbs-positive and -negative individuals

Overall, the demographic characteristics of individuals with and without IFN-I neutralizing autoAbs in the two cohorts were similar (Table 2). The mean age of IFN-I autoAbs-positive individuals in the Danish cohort was 49.9 years, compared to 46.6 years for the IFN-I autoAbs-negative cohort. In the American cohort, the mean age was 42.0 years for the IFN-I autoAbs-positive individuals and 41.7 years for the IFN-I autoAbs-negative individuals. Regarding sex distribution, 1/3 (33%) of the IFN-I autoAbs-positive individuals in the Danish cohort were male and 2/3 (66%) were female. In contrast, the IFN-I autoAbs-negative individuals in the Danish cohort showed the opposite tendency, with 231/315 (73%) being male, 63/315 (20%) being female, and 21/315 (7%) with unreported sex. In the American cohort, all IFN-I autoAbs-positive individuals were male (2/2 [100%]), while the IFN-I autoAbs-negative individuals in the American cohort consisted of 257/290 (88%) male, 31/290 (11%) female, and 2/290 (1%) with unreported sex. In both the Danish and the American cohorts, there was no obvious difference between median CD4^+^ T cell counts between IFN-I autoAbs-positive and IFN-I autoAbs-negative individuals. In the Danish cohort, two of the three IFN-I autoAbs-positive individuals had CD4^+^ T cell counts within the normal range (550 and 830 cells/μl, respectively) (18), while one individual had a CD4^+^ T cell count below the normal range (310 cells/μl). The CD4^+^ T cell counts of IFN-I autoAbs-negative individuals in this cohort varied, with a median of 670 cells/μl (133–2,143), reflecting a generally preserved CD4^+^ T cell count in the majority of individuals. In the American cohort, the two IFN-I autoAbs-positive individuals had CD4^+^ T cell counts of 408 and 741 cells/μl, respectively, thus one individual exhibiting a count just below the normal range and the other within the normal range. The IFN-I autoAbs-negative individuals in the American cohort had a median CD4^+^ T cell count of 469 cells/μl (9–1,530 cells/μl), which was slightly below the normal range of 550 cells/μl. Regarding treatment, all three IFN-I autoAbs-positive Danish individuals (100%) were receiving ART, compared to 257/315 (81.6%) IFN-I autoAbs-negative individuals. In contrast, all individuals in the American cohort, both IFN-I autoAbs-positive and IFN-I autoAbs-negative individuals, were ART-naive, in accordance with this cohort consisting of only ART-naive individuals.

**Table 2.** Demographics of IFN-I autoAbs-positive and autoAbs-negative individuals from the Danish and American cohorts

​	Danish cohort: IFN-I autoAbs-positive individuals	Danish cohort: IFN-I autoAbs-negative individuals	American cohort: IFN-I autoAbs-positive individuals	American cohort: IFN-I autoAbs-negative individuals
**Number of individuals**	3	315	2	290
**Mean age, years [range]**	49.9 [29–67]	46.6 [18–79]	42.0 [28–56]	41.7 [20–76]
**Sex**				
Male	1/3 (33%)	231/315 (73%)	2/2 (100%)	257/290 (88%)
Female	2/3 (66%)	63/315 (20%)	0/2 (0%)	31/290 (11%)
Unknown	0/3 (0%)	21/315 (7%)	0/2 (0%)	2/290 (1%)
**Median CD4** ^**+**^ ** count (cells/μl) [range]**	550 [310–830]	670 [133–2,143]	574.5 [408–741]	469 [9–1,530]
**ART-treated**	3/3 (100%)	257/315 (82%)	0/2 (0%)	0/290 (0%)
**ART-naive**	0/3 (%)	58/315 (18%)	2/2 (100%)	290/290 (100%)

### Clinical history of IFN-I autoAbs-positive individuals with HIV-1

We next investigated the clinical history of the five individuals with IFN-I neutralizing autoAbs, in terms of severe viral diseases and virus-induced cancers, appearing both before and after the detection of IFN-I neutralizing autoAbs (Table 3). Participant (P)1 (Danish cohort) was a 67-year-old male on ART with a CD4^+^ T cell count of 550 cells/μl and co-infected with hepatitis C virus (HCV) for which he was treated with pegylated IFN-α (Pegasys) for 24 wk in the past. At the time of plasma sample collection, he harbored IFN-I neutralizing autoAbs against IFN-ω at 1 ng/ml and 200 pg/ml. He was diagnosed with rectal cancer 17 years prior to the detection of IFN-I neutralizing autoAbs. Four years after the detection of IFN-I neutralizing autoAbs, he was admitted with severe *Streptococcus bovis* bacteremia, and 11 years after the IFN-I neutralizing autoAbs were detected, the patient died due to hepatocellular carcinoma (HCC) as a consequence of chronic HCV infection. P2 (Danish cohort) was a 29-year-old female on ART with a relatively low CD4^+^ T cell count of 310 cells/μl at the time of plasma collection, where neutralizing autoAbs were detected against IFN-ω at 1 ng/ml and 200 pg/ml. She had a history of human papillomavirus (HPV) infection and was diagnosed with a low-grade squamous intraepithelial lesion 1 year after IFN-I neutralizing autoAbs detection, which progressed to carcinoma in situ 2 years later. 5 years after IFN-I neutralizing autoAbs detection, she was hospitalized with pneumococcal pneumonia. P3 (Danish cohort) was a 52-year-old female on ART with a CD4^+^ T cell count of 830 cells/μl at the time of plasma collection, where neutralizing autoAbs were detected against IFN-ω at 200 pg/ml. She was co-infected with HCV for many years and was treated with IFN-α (Pegasys) for 24 mo in 2002, clearing HCV infection prior to sample collection. P4 (American cohort) was a 56-year-old male with a CD4^+^ T cell count of 741 cells/μl at the time of plasma collection and categorized as a non-controller with a high viral load, although he was considered a long-term non-progressor given his maintenance of a robust CD4^+^ T cell count in the absence of ART. Neutralizing autoAbs were detected against IFN-ω at 1 ng/ml and 200 pg/ml. This individual engaged with the healthcare system sporadically, and despite being prescribed ART was frequently found to be viremic, with multiple admissions for community-acquired pneumonia. Prior to the sample collection date, he had a documented history of genital herpes simplex virus (HSV)-2 infection. After the sample collection date, he experienced episodes of herpes zoster including at least one lesion crossing the midline revealing multi-dermatomal involvement, as well as infections with respiratory syncytial virus, parainfluenza virus, and COVID-19. P5 (American cohort) was a 28-year-old ART-naive male with a CD4^+^ T cell count of 408 cells/μl at the time of plasma collection and categorized as a non-controller with a high viral load. IFN-I neutralizing autoAbs were detected against IFN-ω at 1 ng/ml and 200 pg/ml. This individual has a history of multiple sexually transmitted infections (STIs), as well as an HSV-2 infection in 2018, and was also diagnosed with MPox after sample collection.

**Table 3.** Demographics and clinical history of IFN-I autoAbs-positive individuals

​	Cohort	Age (years)	Sex	HIV treatment status	CD4^+^ count (cells/μl)	IFN-I neutralized	Collection year	Infection history	Prior IFN-α treatment
**P1**	Danish	67	Male	ART	550	IFN-ω 1 ng/ml IFN-ω 200 pg/ml	2009	• Rectal cancer (1992) • HCV co-infection • *Streptococcus bovis* bacteremia (2013) • Deceased: May 2020 • Cause of death: HCC	Yes
**P2**	Danish	29	Female	ART	310	IFN-ω 1 ng/ml IFN-ω 200 pg/ml	2011	• Cervical HPV infection developing into LSIL (2012) and carcinoma in situ (2014) • Hospitalized with pneumococcal pneumonia (2016)	No
**P3**	Danish	52	Female	ART	830	IFN-ω 200 pg/ml	2019	• HCV co-infection (chronic since 2002^a^)	Yes
**P4**	American	56	Male	Naive	741	IFN-ω 1 ng/ml IFN-ω 200 pg/ml	2009	• Frequently hospitalized due to viremic episodes and community acquired pneumonia • History of HSV-2 infection (before 2009) • Episodes of disseminated VZV, RSV, parainfluenza, and mild COVID-19 infections (after 2009)	No
**P5**	American	28	Male	Naive	408	IFN-ω 1 ng/ml IFN-ω 200 pg/ml	2018	• History of several episodes with STIs • Documented HSV-2 outbreak in 2018 • Diagnosed with MPox (after 2018)	No

IFN, interferon; ART, antiretroviral therapy; HCV, hepatitis C virus; HCC, hepatocellular carcinoma; HPV, human papillomavirus; LSIL, low-grade squamous intraepithelial lesion; HSV-2, herpes simplex virus type 2; VZV, varicella-zoster virus; RSV, respiratory syncytial virus; STI, sexually transmitted infection

a Chronic HSV infection cleared after IFN-α treatment.

### Detection of IFN-α autoAbs using enzyme-linked immunosorbent assay (ELISA)

**Figure S2.**
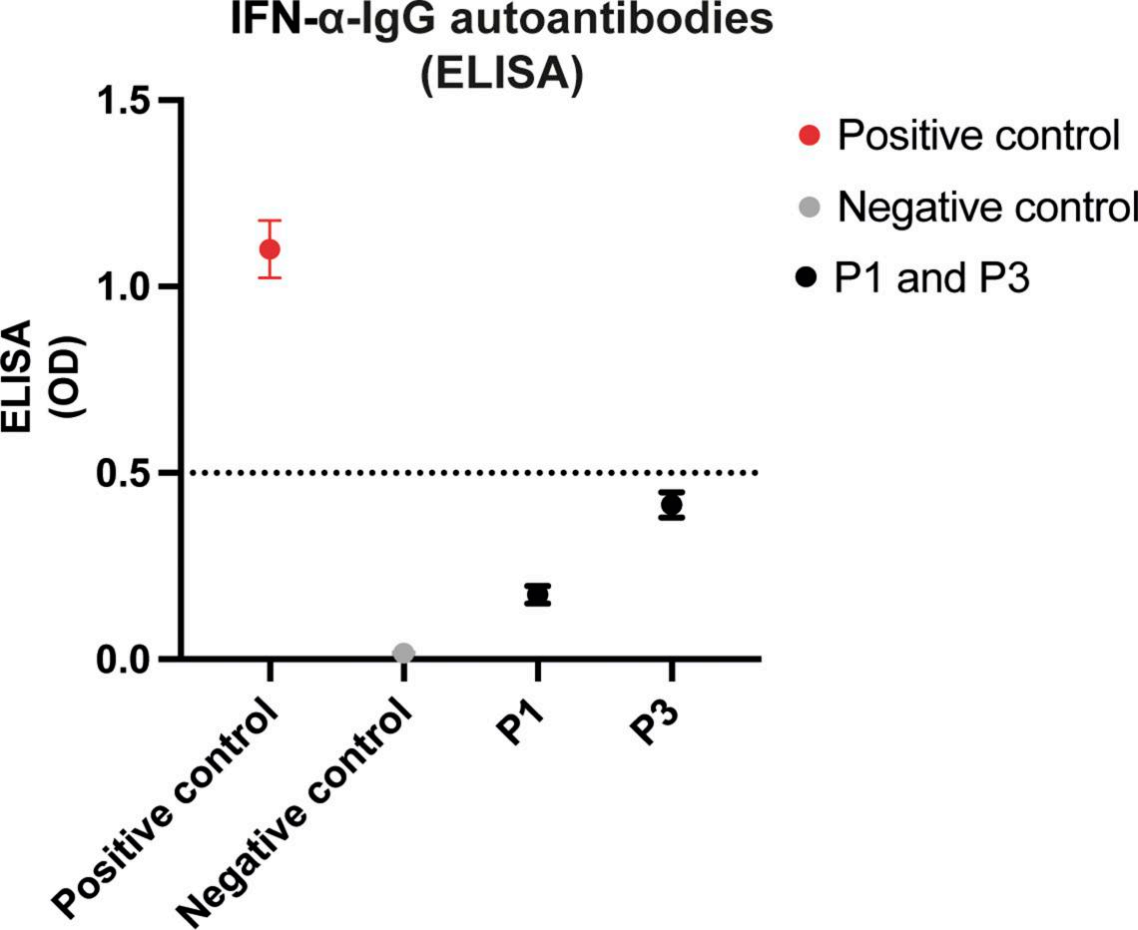
**IFNa-IgG ELISA results from** **P** **1 and** **P** **3.** Plasma samples from P1 and P3 in the Danish cohort were evaluated for the presence of anti-IFN-α IgG autoAbs by ELISA. Absorbance values are shown for each patient, alongside representative positive and negative control samples. The dotted line indicates the assay cut-off for positivity. Positive control (red), negative control (grey), and patient samples (black) are plotted as means with ranges.

P1 and P3 both had a history of IFN-α treatment for chronic hepatitis C infection prior to the detection of IFN-ω neutralizing autoAbs. However, neither of these patients exhibited IFN-α autoAbs when assessed by the neutralization firefly/renilla luciferase assay. To further evaluate the presence of potential non-neutralizing IFN-α autoAbs, an IFN-α ELISA was performed. Both P1 and P3 showed optical density (OD) values below the cut-off of 0.5 (Fig. S2), demonstrating absence of detectable IFN-α autoAbs consistent with the absence of IFN-α neutralizing activity observed.

### Clinical data from the Danish and American cohorts

For the American cohort, medical files were assessed for HCV status, prior IFN-α treatment, and documented herpes zoster virus infection at the time of sampling for all participants. Among the 292 individuals, 70 (24.0%) were HCV antibody positive, 174 (59.6%) were negative, and 48 (16.4%) had unknown status. Only one individual had received prior IFN-α treatment; this participant did not exhibit neutralizing autoAbs against any of the IFN conditions tested. A documented history of herpes zoster was noted for 77 participants (26.4%), while this information was unavailable for the remaining individuals (Table S1).

For the Danish cohort, a large proportion of the samples included were from studies where patients with active or chronic HCV infection, defined by detectable HCV RNA in plasma, were excluded prior to enrollment and plasma collection. Therefore, 148 of the 318 individuals (46.5%) are known to not have active or chronic HCV infection. For the remaining part of the Danish cohort no patients with active HCV infection were included whereas chronic infection could not be entirely excluded but, in that case, would be estimated to affect <5% of the cohort. Information on previous IFN-α treatment was not available for this cohort.

### IFN-I neutralization results from longitudinal samples

**Figure S3.**
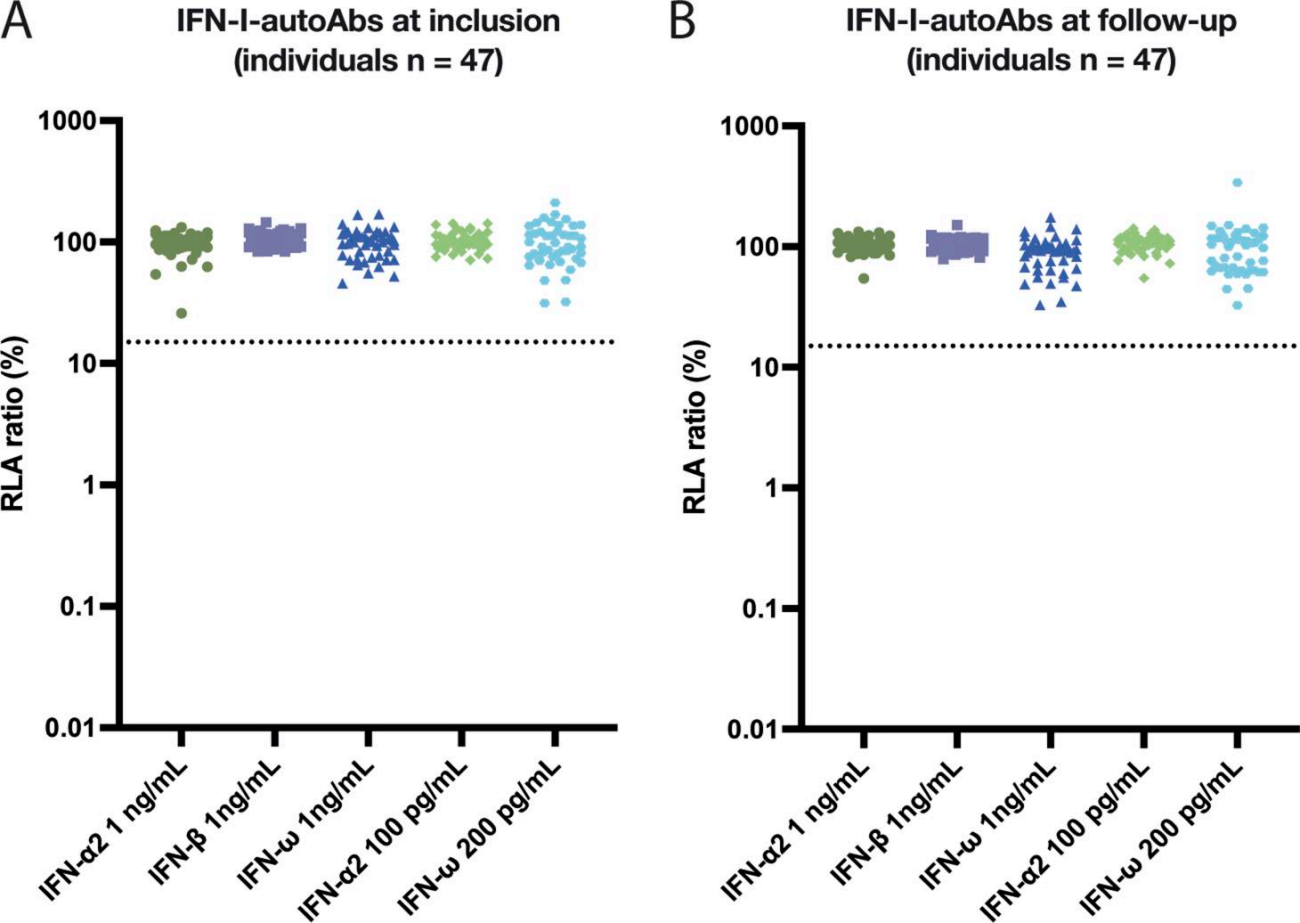
**Longitudinal measurements of IFN-I neutralizing autoAbs in 47 individuals in the Danish cohort. (A)** Measurement of IFN-I neutralizing autoAbs plotted as normalized relative luciferase activity after baseline correction (RLA ratio). **(A and B)** A sub cohort of 47 individuals with HIV-1 with longitudinal plasma samples, including an initial sample taken before a predefined time window (A) and a follow-up sample at a later time point following investigational product treatment (B).

**Figure S4.**
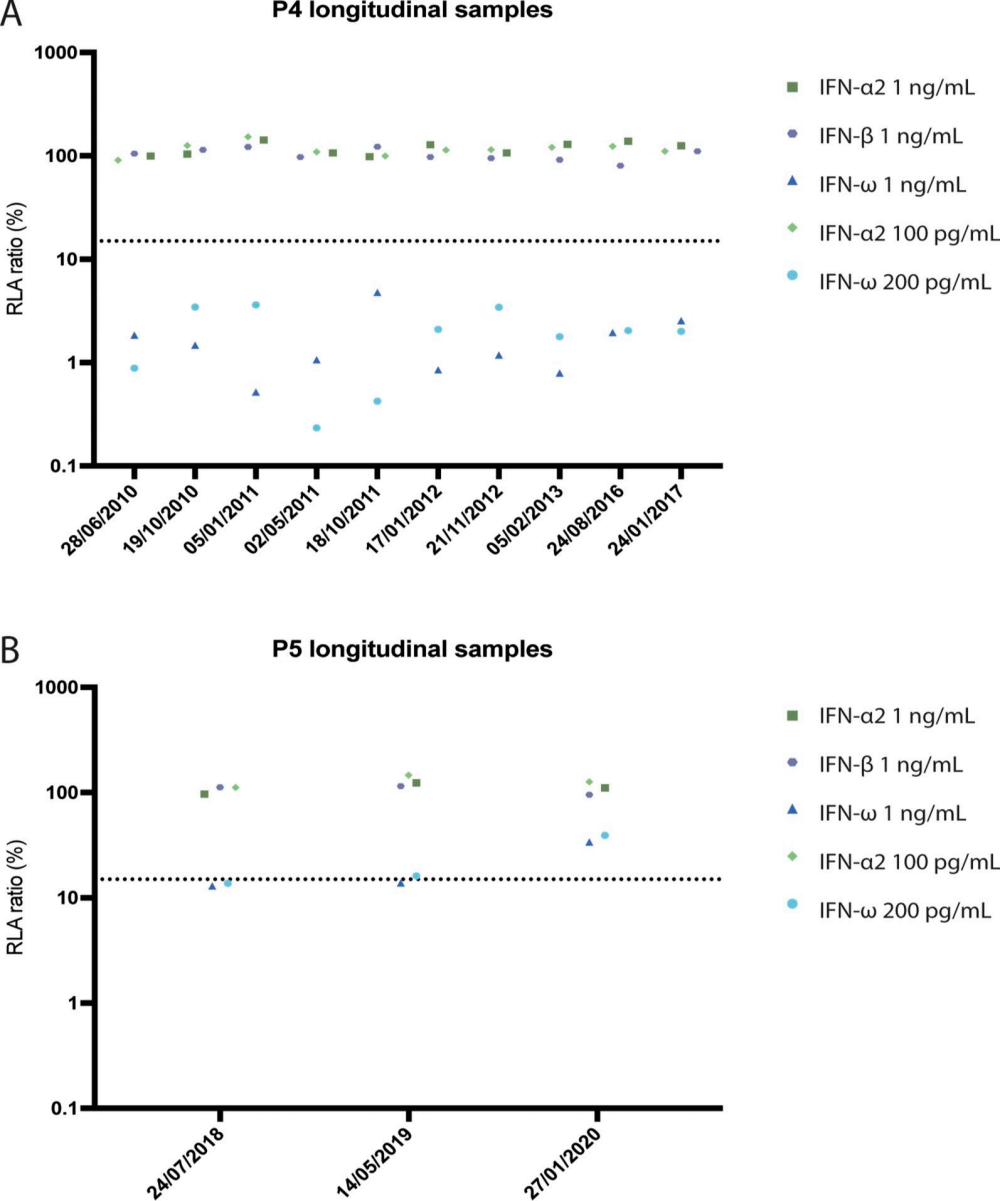
**Longitudinal measurements of IFN-I neutralizing autoAbs in participants P4 and P5.** Measurement of IFN-I neutralizing autoAbs plotted as normalized relative luciferase activity after baseline correction (RLA ratio) for longitudinal plasma samples from P4 (A) and P5 (B). **(A)** 10 plasma samples from P4 were analyzed over a 7-year period (2010–2017), after the initial detection of IFN-I neutralizing autoAbs in 2009. **(B)** Three plasma samples from P5 were analyzed over a 2-year period (2018–2020), following the initial detection of IFN-I neutralizing autoAbs in 2018.

Longitudinal samples from 47 individuals in the Danish cohort were tested for IFN-I neutralizing autoAbs against all five IFN-I conditions as previously mentioned. All individuals received ART at the time of the initial plasma sample, and all individuals received an investigational product treatment for a predefined time period, depending on the original study project from AUH. These individuals have a mean age of 44.3 (±9.2) and a distribution of 38 males, 6 females, and 3 with unknown sex. No IFN-I neutralizing autoAbs were detected in any of these follow-up samples (Fig. S3, A and B), aligning with prior findings that these individuals lacked detectable IFN-I neutralizing autoAbs in the initial samples tested. In the American cohort, longitudinal samples from the two individuals with IFN-I neutralizing autoAbs were analyzed for all five IFN-I conditions. For P4, 10 additional plasma samples obtained between the years 2010 and 2017 were available demonstrating IFN-I neutralizing autoAbs against IFN-ω at both 1 ng/ml and 200 pg/ml present in all the samples throughout the years (Fig. S4 A). For P5, three additional plasma samples obtained between 2018 and 2020 were available (Fig. S4 B) revealing that this individual lost neutralization against IFN-ω at both concentrations through the years.

## Discussion

The presence and persistence of IFN-I neutralizing autoAbs in people with HIV and the potential association with immune pathogenesis, risk of opportunistic infections, and cancer remain largely unknown. In the present study, we found a combined prevalence of IFN-I neutralizing autoAbs of 0.82% in cohorts with a mean age of 49.9 and 42.7 years at the time of sampling, and the majority being males, with 78.1 and 89.0% for the Danish and American cohorts, respectively. This observation aligns with findings from the general healthy population, in which 0.3–1% of individuals below 65 years have IFN-I neutralizing autoAbs, with IFN-ω being the most common (5). In 2022, a high frequency (87.5%) of IFN-I neutralizing autoAbs was shown in a small cohort (eight individuals) of SARS-CoV-2 and HIV-1 co-infected individuals on ART, whereas no IFN-I neutralizing autoAbs were detected in the control group consisting of individuals with HIV-1 infection but without SARS-CoV-2 co-infection (19). Our findings of similar levels of IFN-I neutralizing autoAbs in the two HIV-1 cohorts compared with the background population indicate that the development of COVID-19, rather than HIV-1 status, was related to the presence of IFN-I neutralizing autoAbs as previously demonstrated (4). A small cross-sectional exploratory study from 2023 including 60 patients with HIV and opportunistic infections, including *Mycobacterium tuberculosis*, non-tuberculous mycobacteria, or active CMV infection, reported that 11% were positive for IFN-I autoAbs (against IFN-α or IFN-ω) measured by ELISA (20). Notably, although most of these individuals were receiving ART, only less than half (41%) had undetectable and fully suppressed plasma HIV-1 RNA (<50 copies/ml) at the time of IFN‐I autoAbs testing (20). The discrepancy between this high prevalence of IFN-I autoAbs in this cross-sectional study compared with ours might be explained by the fact that IFN-I autoAbs were detected by ELISA, which may be more likely to detect autoAbs without neutralizing capacity, and not by a functional neutralization assay (6, 10). In the study by Imberti et al. the presence of IFN-I autoAbs did not correlate with CD4^+^ T cell count or viral load at time of measurement but showed a positive correlation with a high viral load at HIV-1 diagnosis (20). We also did not observe a correlation between CD4^+^ T cell count and the presence of IFN-I neutralizing autoAbs, with 3/5 (60%) IFN-I neutralizing autoAbs positive individuals having CD4^+^ T cell counts within the normal range (500–1,400 cells/μl). However, the two IFN-I–neutralizing individuals from the American cohort exhibited high viral loads at the time of plasma collection, together with previous findings indicating that persistent viremia might possibly represent a driver of autoAbs development.

A seminal study by Hale and associates from 2024, based on data from 1,876 individuals with HIV from the Swiss HIV Cohort, reported that 1.9% of individuals developed IFN-I autoAbs using a multiplex bead-based assay to screen samples for IgG autoAbs against IFN-α2, IFN-β, and IFN-ω, whereas 1.17% had IFN-I neutralizing autoAbs against these IFN-Is detected via a dual-luciferase reporter assay (17). These individuals with neutralizing autoAbs against at least one IFN-I were divided as follows: 0.58% of 65–69-year-olds (5/857); 1.68% of 70–79-year-olds (14/831); and 1.64% of 80–89-year-olds (3/183) and were largely similar to those previously reported in an otherwise healthy, general population cohort (5). The authors concluded that once developed, these IFN-I neutralizing autoAbs persisted for up to 15 years and were associated with decreased mRNA levels of IFN-stimulated genes in peripheral blood mononuclear cells (PBMCs), increased risk of severe COVID-19, and possibly an age-related loss of self-tolerance. A significant negative association was observed between CMV seroprevalence in individuals with IFN-I autoAbs compared to those without, whereas a significant positive association was observed in relation to prior herpes zoster events. No associations were observed between the presence of IFN autoAbs and CD4^+^ T cell count, HIV-1 RNA levels, or common opportunistic bacterial or fungal infections. Moreover, the fraction of IFN-I neutralizing autoAbs positivity in these virally suppressed people with HIV was largely similar to the general population, although IFN-I neutralizing autoAbs positivity in untreated, viremic individuals was not assessed (17). The present study, which included both ART-treated and ART-naive people with HIV, also showed a similar prevalence of IFN-I neutralizing autoAbs to that of the general population below 65 years of age (5), suggesting that ART status and viremia do not strongly influence IFN-I neutralizing autoAbs development at population level.

We identified five individuals with HIV-1 and IFN-I neutralizing autoAbs who shared an extensive history of viral infections and virus-induced cancer, suggesting a potential link between IFN-I neutralizing autoAbs and recurrent and/or persistent/chronic viral infections in these individuals. For instance, P1 had a longstanding HCV co-infection, for which he was treated with IFN-α. Later, the chronic HCV infection progressed into HCC. P3 similarly had chronic HCV infection for 17 years before plasma collection and received IFN-α treatment. P4 and P5 had recurrent infections with HSV-2, varicella-zoster virus (VZV), respiratory syncytial virus (RSV), and parainfluenza, indicating a potentially impaired innate immune response contributing to viral infection and persistence. Such recurrent HSV-2 and VZV infections are relatively common among people living with HIV. HSV-2 co-infection is particularly prevalent, with global seroprevalence rates often exceeding 50%, although considerable regional variation exists (21, 22). In contrast, a study of women living with HIV in Denmark found that only 2% were HSV-2 positive by PCR testing (23). The incidence of herpes zoster has been estimated to be 10–30 times higher in ART-naive HIV-positive individuals than in HIV-negative controls (24, 25), which aligns with our American cohort, where 26.4% of participants had a documented history of herpes zoster, whereas status was unknown for the remaining 73.6%. P2 had a history of HPV-induced carcinoma in situ as well as hospitalization with pneumococcal pneumonia, which occurred 3 and 5 years after the detection of IFN-I neutralizing autoAbs, respectively. However, people living with HIV generally have an increased risk of developing HPV-associated malignancies independently of IFN-I autoAbs (26, 27).

Notably, none of the five individuals with IFN-I neutralizing autoAbs reported any of the diseases previously associated with IFN-I autoAbs, including severe influenza or severe COVID-19 (4, 6). Longitudinal samples from P4 and P5 revealed that persistence of autoAbs can vary, with P4 maintaining neutralizing autoAbs against IFN-ω over a period of 7 years, whereas P5 appeared to lose detectable neutralizing IFN-ω autoAbs within a 2-year period. This transient nature of autoAbs upon IFN-α treatment was also observed in the Swiss HIV Cohort, in which three patients treated with IFN-α for chronic HCV infection developed autoAbs to IFN-α and in one of these also to IFN-ω. In two of these patients, IFN-I autoAbs disappeared over time, whereas in one individual, they lasted for more than two decades (17).

Our data demonstrate that both ART-treated virally suppressed and ART-naive people with HIV overall have a prevalence of IFN-I neutralizing autoAbs comparable to the general population (5). In addition, our study provides insight into individual cases, suggesting a potential association between IFN-I neutralizing autoAbs and an increased risk of viral opportunistic infections and virus-related cancers, although this is difficult to demonstrate with certainty based on the current dataset. Notably, the pronounced infection susceptibility in the five individuals with IFN-I neutralizing autoAbs was not associated with a decreased CD4^+^ T cell count, thereby arguing against a cellular immunodeficiency as a potential contributing factor to the infection and cancer susceptibility. We cannot exclude that IFN-I neutralizing autoAbs in the two individuals (P1 and P3) with HCV-co-infection might have been induced by IFN-α treatment as previously reported (17, 28). Within the American cohort, only one patient (1/302) received IFN-α treatment before sampling, and this subject did not show evidence of neutralizing autoAbs against any of the IFN-subtypes tested. For the Danish cohort, information on previous IFN-α treatment for chronic HCV infection is unavailable for autoAbs negative individuals. Notably, we detected IFN-ω neutralizing autoAbs as opposed to IFN-α neutralizing autoAbs reported in the study by Hale and associates, questioning the association between IFN-α treatment and development of IFN-α autoAbs, or suggesting cross-reactivity between IFN-α and IFN-ω autoAbs. Consistent with these data, it has been demonstrated that patient-derived monoclonal autoAbs can neutralize several IFN-α subtypes and IFN-ω (29). However, samples from P1 and P3 harboring neutralizing IFN-ω but not IFN-α autoAbs were, additionally, tested by ELISA which also did not detect any IFN-α autoAbs. Therefore, the emergence of neutralizing IFN-ω autoAbs in the absence of detectable IFN-α autoAbs challenges the theory that IFN-α therapy alone drives the generation of the neutralizing IFN-ω autoAbs, or reflects that any IFN-α autoAbs triggered byprior IFN-α treatment may have been transient and disappeared at the time of sampling.

Whether IFN-I neutralizing autoAbs may have been present at the time of HCV infection and could have contributed to chronicity and progression to HCC remains unknown and cannot be resolved here due to the lack of plasma prior to or at the time of HCV transmission and early infection. Based on these data together with previous findings (2, 18, 30, 31), we suggest that presence of neutralizing IFN autoAbs might have preceded and predisposed to infections with herpesviruses and HPV-induced malignancy in these individuals. In contrast, the natural history of HCV infection and IFN treatment for this condition may likely have preceded the development of neutralizing IFN autoAbs as previously documented (17).

Collectively, we studied two Danish and American cohorts of individuals with HIV-1 infection, mainly below 65 years, who were either ART-treated or ART-naive, and found that 5/610 (0.82%) individuals harbored IFN-I neutralizing autoAbs for at least one IFN-I condition. At cohort level, this is not a significantly increased fraction of IFN-I neutralizing autoAbs compared to the general population. We did not observe any clear correlations of IFN-I neutralizing autoAbs with ART-treatment, CD4^+^ T cell count, or other parameters available to us. However, given the substantial disease burden of those five individuals with IFN-I neutralizing autoAbs, including recurrent HSV and VZV infections and HPV-related carcinoma in situ (and possibly chronic HCV with HCC), we suggest that the presence of IFN-I neutralizing autoAbs in each of these individuals may contribute to overall increased infection susceptibility and virus-induced cancer, possibly in the context of affected cellular immunity and T cell exhaustion. Further studies are needed to more precisely investigate the presence and impact of IFN-I neutralizing autoAbs on viral and non-viral autoimmune and malignant disease presentations/pathologies in people infected with HIV.

## Materials and methods

### Study population

Plasma samples were analyzed from two independent Danish and American cohorts of individuals with HIV. Danish cohort: Adult individuals with HIV (≥18 years old) were included from previous studies, from which samples were still available. The cohort comprises samples collected from nine different HIV studies conducted at AUH, Aarhus, Denmark (32, 33, 34, 35, 36, 37, 38, 39, 40). Samples were collected between January 21, 2008, and December 2, 2021, from 318 individuals with HIV. These sample collections, previously obtained from clinical trials at AUH, comprising both ART-treated individuals (32, 33, 35, 36, 37, 38, 39, 40) and ART-naive individuals (33, 34, 36). For 47 individuals, longitudinal samples were available, where all individuals were receiving ART prior to the initial sample collection. Following this, participants received an investigational product treatment as part of clinical trials for a defined period, before the longitudinal samples were obtained. The treatments included: 3BNC117 (a broadly neutralizing and highly potent anti-HIV-1 antibody) and/or romidepsin (a histone deacetylase [HDAC] inhibitor) (*n* = 13) (32, 34); Vacc-4x (a peptide-based therapeutic HIV-1 vaccine), recombinant human granulocyte-macrophage colony-stimulating factor, and romidepsin (*n* = 15) (37); panobinostat (an HDAC inhibitor that induces viral production in latently infected cells in vitro) (*n* = 10) (40); or MGN1703 (a Toll-like receptor 9 agonist) (*n* = 6) (39). American cohort: Plasma samples from 292 ART-naive adult individuals with HIV (≥18 years old), were obtained from the observational SCOPE cohort at UCSF. Plasma was collected over a span of 18 years (between October 2000 and November 2018). These individuals were classified into three groups based on viral load: non-controllers (*n* = 206), viremic controllers (who had consistent viral loads >50 copies/ml and <5,000 copies/ml, despite no treatment with ART) (*n* = 71), and elite controllers (consistently low or undetectable viral load over time despite no treatment with ART) (*n* = 15). For two individuals in this cohort, longitudinal samples were studied. All plasma samples were stored at −80°C until analysis.

### Luciferase-based neutralization assay

The ability of plasma samples to neutralize IFN-I was assessed as previously described (5, 9). Briefly, plasma samples were heat-inactivated in a PCR cycler at 56°C for 30 min prior to use. HEK-293T cells were seeded in 96-well plates with 15,000 cells per well in 100 μl Dulbecco’s modified Eagle’s medium (DMEM) (#D6429; Sigma-Aldrich) supplemented with 10% fetal calf serum (FCS) (#F7524; Sigma-Aldrich) and 1% penicillin-streptomycin (pen-strep) (#10378-016; Thermo Fisher Scientific, 100 IU/ml). After overnight incubation, cells were transfected with 98 ng pGL4.45 (ISRE firefly luciferase) and 2 ng of a Renilla luciferase-encoding plasmid per well using XtremeGENE 9 DNA Transfection Reagent (#06365787001; Sigma-Aldrich) in OptiMEM (#31-985-070; Thermo Fischer Scientific), at a transfectant:DNA ratio of 1:3. The following day, the supernatant was removed, and cells were incubated for 16 h in 50 μl DMEM containing the indicated IFN-I (see below), 2% FCS, 1% pen-strep, and 10% plasma. Firefly and Renilla luciferase activities were measured using the Dual-Luciferase Reporter Assay System (#E2980; Promega). Unglycosylated IFN-I was tested at 1 ng/ml and 100 pg/ml for IFN-α2 (130-093-874; Miltenyi Biotec) and at 1 ng/ml and 200 pg/ml for IFN-ω (300-02j-100UG; Peprotech). Glycosylated IFN-I was tested at 1 ng/ml for IFN-β (300-02BC-100UG; PeproTech). Luminescence was measured using a Synergy LX multi-mode reader (BioTek). Relative luciferase activity (RLA) was calculated (firefly luciferase activity divided by Renilla luciferase activity). To remove the baseline signal, RLA values were further normalized to the RLA value of a control sample from the same plate not stimulated with IFN-I. This baseline-corrected RLA value was then normalized to the median induction level of the plate of non-neutralizing samples. The results are expressed as a percentage as the normalized RLA after baseline correction, % (RLA ratio). Samples were considered neutralizing if the RLA ratio fell below 15% of the median control value from the same experiment. For each plate, plasma from two healthy adult blood donors was also collected to serve as negative controls. Plasma from an APS-1 patient known to have broad neutralization status against all IFN-I conditions tested was used as a positive control.

### ELISA

Corning Costar Assay Plates (CLS3474-24EA; Sigma-Aldrich) were coated overnight at 4°C with human IFN-α2 (130-093-874; Miltenyi Biotec) at a concentration of 1 μg/ml. The plates were washed three times with phosphate buffered saline (PBS) (D8537-500ML; Sigma-Aldrich) and blocked with 2% bovine serum albumin (BSA) in PBS for 1 h at room temperature. After blocking, the plates were washed three times with washing buffer (PBS containing 0.005% Tween20). Patient samples, diluted 1:50 in 2% BSA and 0.005% Tween-20 in PBS, were added and incubated for 2 h at room temperature. The ELISA was performed once in duplicates on plasma from two patients positive for IFN-ω neutralizing autoAbs. Following three additional washes, horseradish peroxidase–conjugated goat anti-human IgG (Nordic-Mubio, Netherlands) was added at 1 μg/ml for detection and incubated for 1 h. The plates were washed three times before addition of tetramethylbenzidin (TMB) one solution substrate (G7431; Promega) for 5 min. The reaction was stopped with 1M H2SO4 (1603131003; Sigma-Aldrich), and absorbance was measured using a Synergy LX multi-mode reader (BioTek) as the difference between readings at 450 and 630 nm. A cut-off value of 0.5 (blank-corrected OD450–OD630) was used to determine autoAbs positivity. This threshold was established based on two larger studies from the group of J.-L. Casanova, University of Paris, Imagine Institute, France (4, 5), which demonstrated that samples with IFN autoAbs levels below 0.5 lacked neutralizing capacity, whereas those exceeding 0.5 were highly likely to possess neutralizing activity. Anti-hIFN-α-IgG (Invivogen, mabg-hifna3) was included as a positive control, and 2% BSA and 0.005% Tween-20 in PBS served as a negative control.

### Statistics

Mean values with SD and median values were used to describe numerical variables, while counts and percentages were used for categorical variables. Data were analyzed in R (v.4.2.0) using R studio. Figures were prepared with GraphPad Prism (v. 10.3.1, GraphPad Software) and Adobe Illustrator (v. 29.3.1, Adobe Inc.).

### Ethics

All participants for the Danish cohort provided informed consent and the study was approved by the Danish Health Research Ethics Committee with approvals (CLEAR: 1-10-72-98-12; REDUC: M-2013–364–13; TEACH: 1-10-72-10-15; DYNAMO: 1-10-72-311-13; ROADMAP: 1-10-72-355-15; eCLEAR: 1-10-72-110-16; TITAN: 1-10-72-292-18; HIPAVAC: M20110108; ITAP: M-20070135). The study on the American cohort was approved by the UCSF Institutional Review Board. All participants provided written informed consent for their biospecimens to be used to support HIV research.

### Online supplemental material

Fig. S1 shows validation of the neutralization assay using IFN-I autoAb-positive and -negative control plasma samples. Fig. S2 shows IFNα-IgG-positive ELISA results from P1 and P3. Fig. S3 shows longitudinal measurements of IFN-I neutralizing autoAbs from 47 individuals in the Danish cohort. Fig. S4 shows longitudinal measurements of IFN-I neutralizing autoAbs in participants P4 and P5. Table S1 shows clinical data from the American cohort.

## Supplementary Material

Table S1 shows clinical data from the American cohort.

## Data Availability

All relevant data that can be shared can be found in supplemental material. Additional data are available from the corresponding author upon reasonable request from health care professionals and within national General Data Protection Regulation rules and regulations.

## References

[1] Bastard, P., A. Gervais, T. Le Voyer, Q. Philippot, A. Cobat, J. Rosain, E. Jouanguy, L. Abel, S.-Y. Zhang, Q. Zhang, . 2024. Human autoantibodies neutralizing type I IFNs: From 1981 to 2023. Immunol. Rev. 322:98–112. 10.1111/imr.13304 38193358 PMC10950543

[2] Mogensen, K.E., P. Daubas, I. Gresser, D. Sereni, and B. Varet. 1981. Patient with circulating antibodies to alpha-interferon. Lancet. 2:1227–1228. 10.1016/s0140-6736(81)91460-4 6171694

[3] Pozzetto, B., K.E. Mogensen, M.G. Tovey, and I. Gresser. 1984. Characteristics of autoantibodies to human interferon in a patient with varicella-zoster disease. J. Infect. Dis. 150:707–713. 10.1093/infdis/150.5.707 6238105

[4] Bastard, P., L.B. Rosen, Q. Zhang, E. Michailidis, H.-H. Hoffmann, Y. Zhang, K. Dorgham, Q. Philippot, J. Rosain, V. Béziat, . 2020. Autoantibodies against type I IFNs in patients with life-threatening COVID-19. Science. 370:eabd4585. 10.1126/science.abd4585 32972996 PMC7857397

[5] Bastard, P., A. Gervais, T. Le Voyer, J. Rosain, Q. Philippot, J. Manry, E. Michailidis, H.-H. Hoffmann, S. Eto, M. Garcia-Prat, . 2021. Autoantibodies neutralizing type I IFNs are present in ∼4% of uninfected individuals over 70 years old and account for ∼20% of COVID-19 deaths. Sci. Immunol. 6:eabl4340. 10.1126/sciimmunol.abl4340 34413139 PMC8521484

[6] Zhang, Q., A. Pizzorno, L. Miorin, P. Bastard, A. Gervais, T. Le Voyer, L. Bizien, J. Manry, J. Rosain, Q. Philippot, . 2022. Autoantibodies against type I IFNs in patients with critical influenza pneumonia. J. Exp. Med. 219:e20220514. 10.1084/jem.20220514 36112363 PMC9485705

[7] Alotaibi, F., N.K. Alharbi, L.B. Rosen, A.Y. Asiri, A.M. Assiri, H.H. Balkhy, M. Al Jeraisy, Y. Mandourah, S. AlJohani, S. Al Harbi, . 2023. Type I interferon autoantibodies in hospitalized patients with Middle East respiratory syndrome and association with outcomes and treatment effect of interferon beta-1b in MIRACLE clinical trial. Influenza Other Respir. Viruses. 17:e13116. 10.1111/irv.13116 36960162 PMC10028524

[8] Bastard, P., E. Michailidis, H.-H. Hoffmann, M. Chbihi, T. Le Voyer, J. Rosain, Q. Philippot, Y. Seeleuthner, A. Gervais, M. Materna, . 2021. Auto-antibodies to type I IFNs can underlie adverse reactions to yellow fever live attenuated vaccine. J. Exp. Med. 218:e20202486. 10.1084/jem.20202486 33544838 PMC7871457

[9] Gervais, A., F. Rovida, M.A. Avanzini, S. Croce, A. Marchal, S.C. Lin, A. Ferrari, C.W. Thorball, O. Constant, T. Le Voyer, . 2023. Autoantibodies neutralizing type I IFNs underlie West Nile virus encephalitis in approximately 40% of patients. J. Exp. Med. 220:e20230661. 10.1084/jem.20230661 37347462 PMC10287549

[10] Gervais, A., A. Marchal, A. Fortova, M. Berankova, L. Krbkova, M. Pychova, J. Salat, S. Zhao, N. Kerrouche, T. Le Voyer, . 2024. Autoantibodies neutralizing type I IFNs underlie severe tick-borne encephalitis in approximately 10% of patients. J. Exp. Med. 221:e20240637. 10.1084/jem.20240637 39316018 PMC11448868

[11] Gervais, A., P. Bastard, L. Bizien, C. Delifer, P. Tiberghien, C. Rodrigo, F. Trespidi, M. Angelini, G. Rossini, T. Lazzarotto, . 2024. Auto-Abs neutralizing type I IFNs in patients with severe Powassan, Usutu, or Ross River virus disease. J. Exp. Med. 221:e20240942. 10.1084/jem.20240942 39485284 PMC11533500

[12] Duncan, C.J.A., R.E. Randall, and S. Hambleton. 2021. Genetic lesions of type I interferon signalling in human antiviral immunity. Trends Genet. 37:46–58. 10.1016/j.tig.2020.08.017 32977999 PMC7508017

[13] Hale, B.G. 2023. Autoantibodies targeting type I interferons: Prevalence, mechanisms of induction, and association with viral disease susceptibility. Eur. J. Immunol. 53:e2250164. 10.1002/eji.202250164 37027328

[14] Meager, A., K. Visvalingam, P. Peterson, K. Möll, A. Murumägi, K. Krohn, P. Eskelin, J. Perheentupa, E. Husebye, Y. Kadota, and N. Willcox. 2006. Anti-interferon autoantibodies in autoimmune polyendocrinopathy syndrome type 1. PLoS Med. 3:e289. 10.1371/journal.pmed.0030289 16784312 PMC1475653

[15] Le Voyer, T., A.V. Parent, X. Liu, A. Cederholm, A. Gervais, J. Rosain, T. Nguyen, M. Perez Lorenzo, E. Rackaityte, D. Rinchai, . 2023. Autoantibodies against type I IFNs in humans with alternative NF-kappaB pathway deficiency. Nature. 623:803–813. 10.1038/s41586-023-06717-x 37938781 PMC10665196

[16] Groen, K., R. Kuratli, J. Enkelmann, S. Fernbach, P.D. Wendel-Garcia, W.I. Staiger, M. Lejeune, E. Sauras-Colón, F. Roche-Campo, P. Filippidis, . 2025. Type I interferon autoantibody footprints reveal neutralizing mechanisms and allow inhibitory decoy design. J. Exp. Med. 222:e20242039. 10.1084/jem.20242039 40111224 PMC11924951

[17] Fernbach, S., N.K. Mair, I.A. Abela, K. Groen, R. Kuratli, M. Lork, C.W. Thorball, E. Bernasconi, P. Filippidis, K. Leuzinger, . 2024. Loss of tolerance precedes triggering and lifelong persistence of pathogenic type I interferon autoantibodies. J. Exp. Med. 221:e20240365. 10.1084/jem.20240365 39017930 PMC11253716

[18] Busnadiego, I., I.A. Abela, P.M. Frey, D.A. Hofmaenner, T.C. Scheier, R.A. Schuepbach, P.K. Buehler, S.D. Brugger, and B.G. Hale. 2022. Critically ill COVID-19 patients with neutralizing autoantibodies against type I interferons have increased risk of herpesvirus disease. PLoS Biol. 20:e3001709. 10.1371/journal.pbio.3001709 35788562 PMC9286229

[19] Scordio, M., F. Frasca, L. Santinelli, L. Sorrentino, A. Pierangeli, O. Turriziani, C.M. Mastroianni, G. Antonelli, R.P. Viscidi, G. d'Ettorre, and C. Scagnolari. 2022. High frequency of neutralizing antibodies to type I Interferon in HIV-1 patients hospitalized for COVID-19. Clin. Immunol. 241:109068. 10.1016/j.clim.2022.109068 35764258 PMC9233547

[20] Imberti, L., P. Magro, A. Sottini, V. Quaresima, F. Castelli, and E. Quiros-Roldan. 2023. High frequency of type I interferon auto-antibodies in a group of middle-aged HIV-infected patients: A cross-sectional exploratory study. Immun. Inflamm. Dis. 11:e1056. 10.1002/iid3.1056 38018592 PMC10664390

[21] Barbour, J.D., M.M. Sauer, E.R. Sharp, K.E. Garrison, B.R. Long, H. Tomiyama, K.C. Bassichetto, S.M. Oliveira, M.C. Abbate, D.F. Nixon, and E.G. Kallas. 2007. HIV-1/HSV-2 co-infected adults in early HIV-1 infection have elevated CD4+ T cell counts. PLoS One. 2:e1080. 10.1371/journal.pone.0001080 17957262 PMC2031920

[22] Munawwar, A., and S. Singh. 2016. Human herpesviruses as copathogens of HIV infection, their role in HIV transmission, and disease progression. J. Lab. Physicians. 8:5–18. 10.4103/0974-2727.176228 27013807 PMC4785766

[23] Thorsteinsson, K., S. Ladelund, M. Storgaard, F.F. Rønsholt, I.S. Johansen, G. Pedersen, L.N. Nielsen, J. Bonde, H. Westh, N. Obel, . 2016. Sexually transmitted infections and use of contraceptives in women living with HIV in Denmark - the SHADE cohort. BMC Infect. Dis. 16:81. 10.1186/s12879-016-1412-7 26880101 PMC4754814

[24] Lee, Y.C., C.-C. Hung, M.-S. Tsai, J.-Y. Zhang, P.-Y. Wu, S.-P. Yang, Y.-Z. Luo, H.-Y. Chang, W.-C. Liu, H.-Y. Sun, and S.-C. Chang. 2018. Incidence and risk factors of herpes zoster in human immunodeficiency virus-positive patients initiating combination antiretroviral therapy in Taiwan. J. Microbiol. Immunol. Infect. 51:38–44. 10.1016/j.jmii.2016.04.011 27329131

[25] Buchbinder, S.P., M.H. Katz, N.A. Hessol, J.Y. Liu, P.M. O'Malley, R. Underwood, and S.D. Holmberg. 1992. Herpes zoster and human immunodeficiency virus infection. J. Infect. Dis. 166:1153–1156. 10.1093/infdis/166.5.1153 1308664

[26] Grulich, A.E., M.T. van Leeuwen, M.O. Falster, and C.M. Vajdic. 2007. Incidence of cancers in people with HIV/AIDS compared with immunosuppressed transplant recipients: A meta-analysis. Lancet. 370:59–67. 10.1016/S0140-6736(07)61050-2 17617273

[27] Thorsteinsson, K., M. Storgaard, T.L. Katzenstein, S. Ladelund, F.F. Rönsholt, I.S. Johansen, G. Pedersen, A. Gaardsting, L.N. Nielsen, J. Bonde, and A.-M. Lebech. 2018. Prevalence of cervical, oral, and anal human papillomavirus infection in women living with HIV in Denmark - the SHADE cohort study. J. Clin. Virol. 105:64–71. 10.1016/j.jcv.2018.05.010 29906660

[28] Fink, D.L., D. Etoori, R. Hill, O. Idilli, N. Kartikapallil, O. Payne, S. Griffith, H.F. Bradford, C. Mauri, P.T.F. Kennedy, . 2025. Auto-antibodies against interferons are common in people living with chronic hepatitis B virus infection and associate with PegIFNalpha non-response. JHEP Rep. 7:101382. 10.1016/j.jhepr.2025.101382 40276479 PMC12018104

[29] Meyer, S., M. Woodward, C. Hertel, P. Vlaicu, Y. Haque, J. Kärner, A. Macagno, S.C. Onuoha, D. Fishman, H. Peterson, . 2016. AIRE-deficient patients harbor unique high-affinity disease-ameliorating autoantibodies. Cell. 166:582–595. 10.1016/j.cell.2016.06.024 27426947 PMC4967814

[30] Wang, C., D.E. Potts, B. Sun, M. Toth, B. Ujhazi, S. Sharapova, R. Miller, L. Rosen, M. Yilmaz, K. Larsen, . 2025. Factors associated with and kinetics of anti-IFN-alpha autoantibodies in RAG1/2 deficiency. J. Allergy Clin. Immunol. Glob. 4:100521. 10.1016/j.jacig.2025.100521 40697949 PMC12281840

[31] Walter, J.E., L.B. Rosen, K. Csomos, J.M. Rosenberg, D. Mathew, M. Keszei, B. Ujhazi, K. Chen, Y.N. Lee, I. Tirosh, . 2015. Broad-spectrum antibodies against self-antigens and cytokines in RAG deficiency. J. Clin. Invest. 125:4135–4148. 10.1172/JCI80477 26457731 PMC4639965

[32] Gruell, H., J.D. Gunst, Y.Z. Cohen, M.H. Pahus, J.J. Malin, M. Platten, K.G. Millard, M. Tolstrup, R.B. Jones, W.D. Conce Alberto, . 2022. Effect of 3BNC117 and romidepsin on the HIV-1 reservoir in people taking suppressive antiretroviral therapy (ROADMAP): A randomised, open-label, phase 2A trial. Lancet Microbe. 3:e203–e214. 10.1016/S2666-5247(21)00239-1 35544074 PMC10293654

[33] Sogaard, O.S., N. Lohse, Z.B. Harboe, R. Offersen, A.R. Bukh, H.L. Davis, H.C. Schønheyder, and L. Østergaard. 2010. Improving the immunogenicity of pneumococcal conjugate vaccine in HIV-infected adults with a toll-like receptor 9 agonist adjuvant: A randomized, controlled trial. Clin. Infect. Dis. 51:42–50. 10.1086/653112 20504165

[34] Gunst, J.D., M.H. Pahus, M. Rosás-Umbert, I.-N. Lu, T. Benfield, H. Nielsen, I.S. Johansen, R. Mohey, L. Østergaard, V. Klastrup, . 2022. Early intervention with 3BNC117 and romidepsin at antiretroviral treatment initiation in people with HIV-1: A phase 1b/2a, randomized trial. Nat. Med. 28:2424–2435. 10.1038/s41591-022-02023-7 36253609 PMC10189540

[35] Gunst, J.D., J.F. Højen, M.H. Pahus, M. Rosás-Umbert, B. Stiksrud, J.H. McMahon, P.W. Denton, H. Nielsen, I.S. Johansen, T. Benfield, . 2023. Impact of a TLR9 agonist and broadly neutralizing antibodies on HIV-1 persistence: The randomized phase 2a TITAN trial. Nat. Med. 29:2547–2558. 10.1038/s41591-023-02547-6 37696935 PMC10579101

[36] Toft, L., M. Storgaard, M. Müller, P. Sehr, J. Bonde, M. Tolstrup, L. Østergaard, and O.S. Søgaard. 2014. Comparison of the immunogenicity and reactogenicity of Cervarix and Gardasil human papillomavirus vaccines in HIV-infected adults: A randomized, double-blind clinical trial. J. Infect. Dis. 209:1165–1173. 10.1093/infdis/jit657 24273179

[37] Leth, S., M.H. Schleimann, S.K. Nissen, J.F. Højen, R. Olesen, M.E. Graversen, S. Jørgensen, A.S. Kjær, P.W. Denton, A. Mørk, . 2016. Combined effect of Vacc-4x, recombinant human granulocyte macrophage colony-stimulating factor vaccination, and romidepsin on the HIV-1 reservoir (REDUC): A single-arm, phase 1B/2A trial. Lancet HIV. 3:e463–e472. 10.1016/S2352-3018(16)30055-8 27658863

[38] Leth, S., R. Nymann, S. Jørgensen, R. Olesen, T.A. Rasmussen, L. Østergaard, P.W. Denton, M. Tolstrup, and O.S. Søgaard. 2016. HIV-1 transcriptional activity during frequent longitudinal sampling in aviremic patients on antiretroviral therapy. AIDS. 30:713–721. 10.1097/QAD.0000000000000974 26595541

[39] Vibholm, L., M.H. Schleimann, J.F. Højen, T. Benfield, R. Offersen, K. Rasmussen, R. Olesen, A. Dige, J. Agnholt, J. Grau, . 2017. Short-course toll-like receptor 9 agonist treatment impacts innate immunity and plasma viremia in individuals with human immunodeficiency virus infection. Clin. Infect. Dis. 64:1686–1695. 10.1093/cid/cix201 28329286 PMC5849129

[40] Rasmussen, T.A., M. Tolstrup, C.R. Brinkmann, R. Olesen, C. Erikstrup, A. Solomon, A. Winckelmann, S. Palmer, C. Dinarello, M. Buzon, . 2014. Panobinostat, a histone deacetylase inhibitor, for latent-virus reactivation in HIV-infected patients on suppressive antiretroviral therapy: A phase 1/2, single group, clinical trial. Lancet HIV. 1:e13–e21. 10.1016/S2352-3018(14)70014-1 26423811

