# Supplementary material for: Autoantibodies neutralizing type 1 interferons in two cohorts of people with HIV
Source: J Hum Immun. 2026 Jan 30;2(2):e20250179. doi: 10.70962/jhi.20250179 (PMC12857536; doi:10.70962/jhi.20250179)
Supplement: Table S1 — shows clinical data from the American cohort. [file jhi_20250179_tables1.docx]

**Supplementary Table 1. Clinical data from the American cohort**

|  | **Positive/Yes** | **Negative/No** | **Unknown** | **Total** |
| --- | --- | --- | --- | --- |
| HCV antibodies prior to or at sampling | 70 (24.0%) | 174 (59.6%) | 48 (16.4%) | 292 |
| Diagnosis with herpes zoster | 77 (26.4%) | - | 215 (73.6%) | 292 |
| IFN-α treatment | 1 (0.3%) | 291 (99.7%) | - | 292 |

HCV, hepatitis C virus; IFN, interferon
